# Unraveling key interactions and the mechanism of demethylation during hAGT-mediated DNA repair *via* simulations

**DOI:** 10.3389/fmolb.2022.975046

**Published:** 2022-09-14

**Authors:** Shruti T. G., Shakir Ali Siddiqui, Kshatresh Dutta Dubey

**Affiliations:** ^1^ Department of Life Sciences, School of Natural Sciences, Shiv Nadar Institution of Eminence Delhi-NCR, Uttar Pradesh, India; ^2^ Department of Chemistry, School of Natural Sciences, Shiv Nadar Institution of Eminence Delhi-NCR, Uttar Pradesh, India

**Keywords:** direct repair, MD simulation, QM/MM calculation, DNA repair enzyme, reaction mechanism

## Abstract

Alkylating agents pose the biggest threat to the genomic integrity of cells by damaging DNA bases through regular alkylation. Such damages are repaired by several automated types of machinery inside the cell. O6-alkylguanine-DNA alkyltransferase (AGT) is an enzyme that performs the direct repair of an alkylated guanine base by transferring the alkyl group to a cysteine residue. In the present study, using extensive MD simulations and hybrid QM/MM calculations, we have investigated the key interactions between the DNA lesion and the hAGT enzyme and elucidated the mechanisms of the demethylation of the guanine base. Our simulation shows that the DNA lesion is electrostatically stabilized by the enzyme and the Arg135 of hAGT enzyme provides the main driving force to flip the damaged base into the enzyme. The QM/MM calculations show demethylation of the damaged base as a three-step process in a thermodynamically feasible and irreversible manner. Our calculations show that the final product forms *via* Tyr114 in a facile way in contrast to the previously proposed Lys-mediated route.

## Introduction

The genomic integrity of a cell is constantly threatened by some extracellular and intracellular chemicals, which can damage the nucleotide base of a DNA by covalently attaching an alkyl group (Tardiff et al., 1994; Daniels and Tainer, 2000; Margison and Santibáñez-Koref, 2002; Daniels et al., 2004; Yi and He, 2013; Chatterjee and Walker, 2017). Such damaged DNA can cause deleterious mutations and cytotoxicity in cells (Dolan et al., 1990; Pegg, 1990; Daniels and Tainer, 2000; Wibley et al., 2000; Margison and Santibáñez-Koref, 2002; Daniels et al., 2004; Fang et al., 2008); therefore, the cell has the ultimate machinery to repair such DNA damage. This repairing machinery is carried out mainly by some proteins and/or enzymes that have evolved particularly for this purpose, mainly *via* three different mechanisms: 1) photolesions through photolyases by UV induction, 2) reversal by O6-alkylguanine-DNA alkyltransferase (AGT), and 3) reversal by AlkB family dioxygenases (Daniels et al., 2004; Yi and He, 2013). However, the last two mechanisms, i.e., the damage reversal by AGT and AlkB enzymes, are a direct DNA repair mechanism through the de-alkylation of the damaged base and are, hence, believed to be the most efficient way to repair DNA lesions (Yi and He, 2013). It is anticipated that the mechanism of the repair is highly correlated with the position of the alkylation attack (Mishina et al., 2006). For example, if alkylation occurs at the N^7^ position of the guanine base, it results in an innocuous lesion that is mostly repaired through depurination.

**FIGURE 1:**
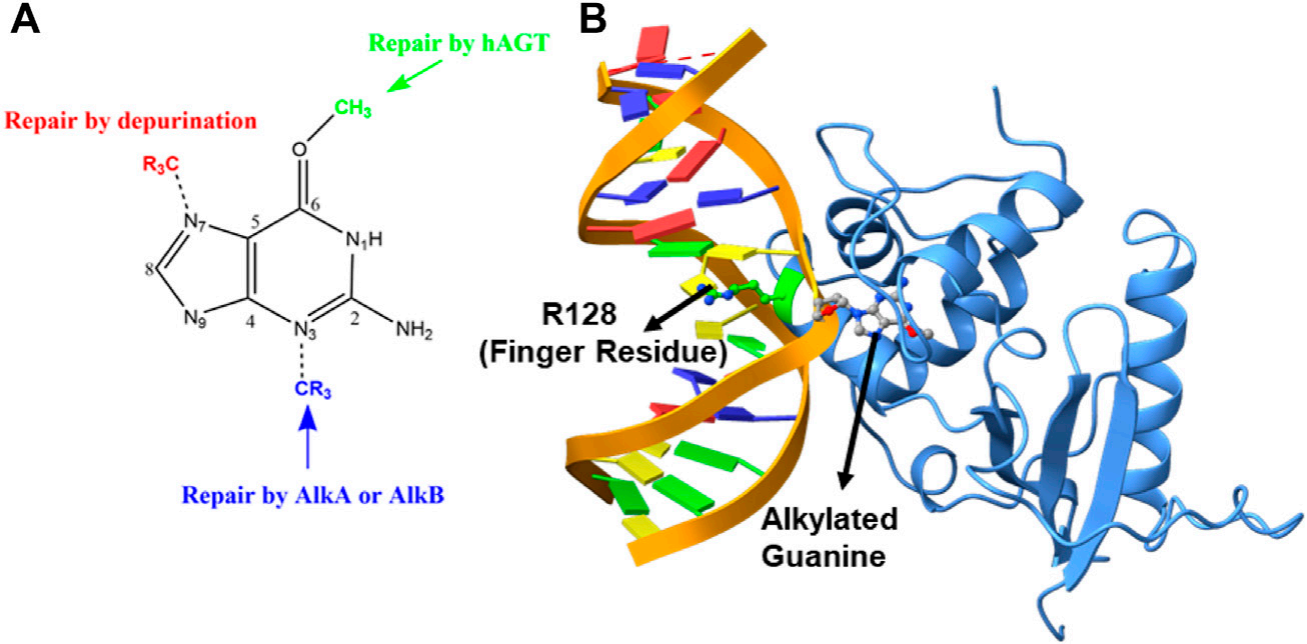
**(A)** DNA repair mechanism based on the position of alkylation. **(B)** Structure of the flipped alkylated guanine in hAGT (PDB ID 1T38).

Similarly, when alkylation occurs at the N^3^ position of the nucleotide base, the resulting lesion blocks DNA replication and is repaired by AlkA or AlkB proteins. The third vulnerable site for alkylation is the oxygen atom (O^6^) of the DNA base to produce the O^6^-methylguanine (Pegg, 1990; Margison and Santibáñez-Koref, 2002) (see Figure 1A). This lesion is believed to be highly mutagenic, and it mispairs with thymine to produce a transition mutation of G: C→T: A during DNA replication (Wibley et al., 2000; Duguid et al., 2005; Tubbs et al., 2007; Yi and He, 2013). These lesions are repaired by the O6-alkylguanine-DNA alkyltransferase (AGT) family of proteins by a suicidal direct repairing mechanism (Dolan et al., 1990; Gerson et al., 1995; Daniels et al., 2000; Daniels and Tainer, 2000; Wibley et al., 2000; Margison and Santibáñez-Koref, 2002; Rasimas et al., 2003; Fang et al., 2008; Yi and He, 2013). In the current work, we have highlighted the key interactions and the mechanism of DNA repair of human AGT protein using MD simulations and hybrid QM/MM calculations.

The crystal structure of the hAGT protein complex with double-strand DNA (dsDNA) carrying the damaged guanine (Daniels et al., 2004) provides crucial insight into base flipping after alkylation. This structure shows the base flipping by a minor grove of the dsDNA (Daniels et al., 2004) and the modified base is inserted into the active site of the protein (Daniels et al., 2004; Fang et al., 2008), which is surrounded by Cys145, Tyr114, Pro140, Ser159, and Tyr158 (Daniels et al., 2000; Daniels and Tainer, 2000; Wibley et al., 2000). Here Cys145 participates directly in the repair by accepting the alkyl group (Daniels and Tainer, 2000; Wibley et al., 2000; Daniels et al., 2004; Fang et al., 2008) while Tyr114 is believed to facilitate a proton transfer in the reaction. An Arg128 residue assigned as the “finger residue” was found to (Figure 1B) be inserted inside the DNA duplex, in place of the damaged, extrahelical base (Daniels and Tainer, 2000; Margison and Santibáñez-Koref, 2002; Daniels et al., 2004; Fang et al., 2008). Interestingly, the role of this “finger residue” is supposed to be instrumental in identifying the damaged base (Tubbs et al., 2007) by sliding over DNA bases (Daniels et al., 2000) by checking the weakened base–base interaction due to the alkylation of the guanine base (sometimes thymine).

The experimental structure of hAGT with the damaged DNA provided a good starting geometry to validate the repair mechanism using computational tools. However, unlike AlkB where the mechanism of DNA repair has been extensively studied (Yi and He, 2013), the repair mechanism by hAGT is relatively less elucidated. Jena et al. (2009) performed a DFT-only study to explore the repair mechanism by hAGT and proposed a three-step pathway for the repair mechanism. According to their study, in the first step, deprotonation of Cys145 occurs *via* a water-mediated mechanism from His146. In the second step, protonation at the N3 position takes place *via* Tyr114, and in the last step, demethylation of guanine occurs through Cys145 (Daniels et al., 2000; Daniels and Tainer, 2000). However, the proposed reaction profile was thermodynamically not feasible since they conducted a DFT-only study without the inclusion of the protein and DNA molecules. Another study by Hou et al. (2010) used a more accurate QM/MM method to explore the repair mechanism of this enzyme. Interestingly, this study shows that the methyl transfer from damaged DNA to cysteine is a reversible process; however, in that case, the “N” of O6-methylguanine is taken in the already protonated state and no calculations had been performed for this investigation. We, therefore, planned to re-investigate the mechanism of the repair by hAGT using extensive MD simulations and hybrid QM/MM calculations. In the present study, we have used a comprehensive MD simulation of the hAGT enzyme with dsDNA bearing alkylated guanine to study the interactions between the enzyme and the modified base and performed hybrid QM/MM calculations to validate the reaction mechanism of the direct DNA repair by hAGT.

## Computational details

We have performed the MD simulations to study the conformational changes and protein–DNA interactions while hybrid QM/MM calculations were performed for the reaction mechanism. The details of each calculation are discussed as follows.

### System setup

The initial coordinate of the hAGT complex with dsDNA was imported from the protein data bank (PDB ID: 1T38) (Daniels et al., 2004). The crystal structure contains an alkylated guanine base flanged out from the DNA strand and buried inside the protein site. The missing residues have been added using the MODELLER program (Webb and Sali, 2017). The parameters for the modified base were prepared using an antechamber module of the Amber MD program of QM-optimized geometry at the HF/6–31 g(d,p) level of the theory. For the protein, we used an Amber ff19SB (Tian et al., 2019) forcefield while for DNA we used a refined Barcelona forcefield implemented in the Amber MD library. A few Na+ ions were added to the protein surfaces to neutralize the total charge of the system depending upon the charge of each complex prepared separately. Finally, the resulting systems were solvated in an octahedral box of an OPC water model each extended up to a minimum cut-off of 10 Å from the protein boundary. pKa of titrable groups were calculated by PropKa and a table for the same is shown in, Supplementary Table S1.

### MD simulations

After proper parameterization of the system, to remove bad contacts, minimization was performed in two stages using a combination of the steepest descent (5,000 steps) and conjugate gradient (5,000 steps) methods. In the first stage, water position and conformations are relaxed keeping the protein fixed. Thereafter, the whole complex was minimized. Subsequently, the system is gently annealed up to 300 K under the NVT ensemble for 50 ps. After that, 1 ns of density equilibration was performed under an NPT ensemble at a target temperature of 300 K and pressure of 1 atm by using a Langevin thermostat (Izaguirre et al., 2001) and Berendsen barostat (Berendsen et al., 1984) with a collision frequency of 2 ps and pressure relaxation time of 1 ps. This 1 ns density equilibration is a weakly restrained MD simulation in which the system is slowly released to achieve uniform density after heating under periodic boundary conditions. Then, after we remove all the restraints applied before, the system gets equilibrated for 3 ns to get a well-settled pressure and temperature for chemical and conformational analyses. Thereafter, a productive MD simulation was performed using the Monte Carlo barostat (Åqvist et al., 2004) for a total of 500 ns for each complex in five subsequent steps of 100 ns starting from the random velocity. During all the MD simulations, covalent bonds containing hydrogens were constrained using the SHAKE (Ryckaert et al., 1977) algorithm, and the Particle Mesh Ewald (PME) (Darden et al., 1993) method was used to treat long-range electrostatic interactions with the cut-off set as 10 Å. All the MD simulations were performed with the GPU version of the AMBER 20 package (Salomon-Ferrer et al., 2013). The MD trajectory analysis was done with the CPPTRAJ (Roe and Cheatham, 2013) module of AMBER 20. The visualization of the MD trajectories was performed by VMD (Humphrey et al., 1996). The binding free energy was calculated using the molecular mechanics generalized born surface area method (MMGBSA), the details and other applications for the nucleic acid complexes have been discussed elsewhere (Chaubey et al., 2012; Sur et al., 2017).

### QM/MM methodology

The mechanism of reaction during base-repairing was calculated using hybrid QM/MM calculations for the representative snapshots from the most populated trajectory of the MD simulations after clustering. The active region in QM/MM calculations in all the systems involves the protein residues and water molecules present within the cutoff of 8 Å from the active oxidant heme. The atoms in the selected “active region” (mainly from the MM part) interact with the QM zone through electrostatic and Van der Waals interactions and the corresponding polarization effects were considered in the subsequent QM/MM calculations. All QM/MM calculations were performed with ChemShell (Sherwood et al., 2003; Metz et al., 2014) by combining the Turbomole (Ahlrichs et al., 1989; Balasubramani et al., 2020) for the QM part and DL_POLY (Smith and Forester, 1996) for the MM part. The MM part was described using the ff19SB forcefield. To account for the polarizing effect of the protein environment on the QM region, an electronic embedding scheme was used. Hydrogen link atoms with the charge shift model were employed for treating QM/MM boundary. During QM/MM geometry optimizations, potential energy surface scanning, and frequency calculations, the QM region was treated using the hybrid B3LYP functional with a def2-SVP basis set. The energies were further corrected with the Grimme dispersion correction. All of the QM/MM transition states were located by relaxed potential energy surface (PES) scans followed by full TS optimizations using the P-RFO (Kästner et al., 2009) optimizer implemented in the HDLC code.

### Free-energy calculations

We executed MMGBSA calculations to determine the free energy of decomposition, to examine the residue-specific interactions with m-GUA (Fogolari et al., 2003; Gohlke and Case, 2004). This approach is based on tried-and-true ideas (Tsui and Case, 2000; Grochowski and Trylska, 2008) that have been effectively applied in many earlier investigations (Xue et al., 2012; Dubey et al., 2013; Dubey et al., 2017; Webb and Sali, 2017; Yang et al., 2018; Siddiqui and Dubey, 2022). In the beginning, we removed all the water molecules and counterions from the trajectory and utilized the solute and solvent’s respective dielectric constants of 1 and 80. Subsequently, for the most populated trajectories, we carried out the MMGBSA calculations.

## Results and discussion

### MD simulations of the hAGT enzyme with the alkylated base after flipping

**FIGURE 2:**
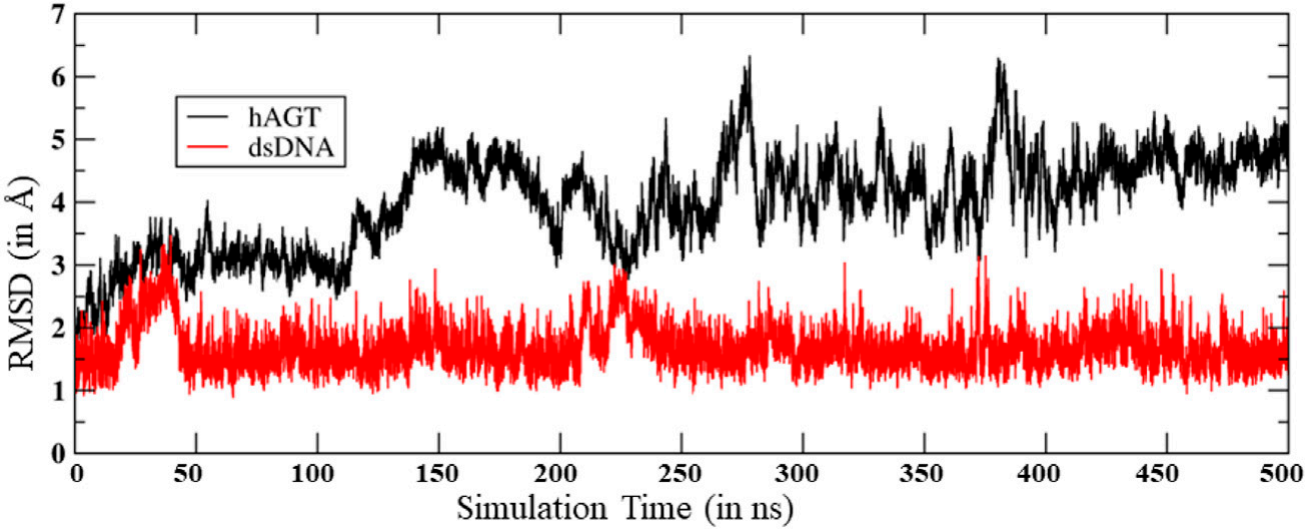
RMS deviation for hAGT and dsDNA during the MD simulations.

To study the mechanisms, we started with the MD simulation of the flipped methylated guanine (m-GUA) with hAGT to investigate the conformational changes, if any. During the entire course of the simulation of 500 ns time, we did not observe many conformational changes in the dsDNA which can be validated by the low root mean square (RMS) deviation as shown in Figure 2 relative to the enzyme. We found that most of the deviation in the enzymatic site occurs due to several loop regions of the hAGT enzyme. To further validate the flexibility, we also supplemented our results with the root mean square fluctuations (RMSF) for residues during the simulations.

**FIGURE 3:**
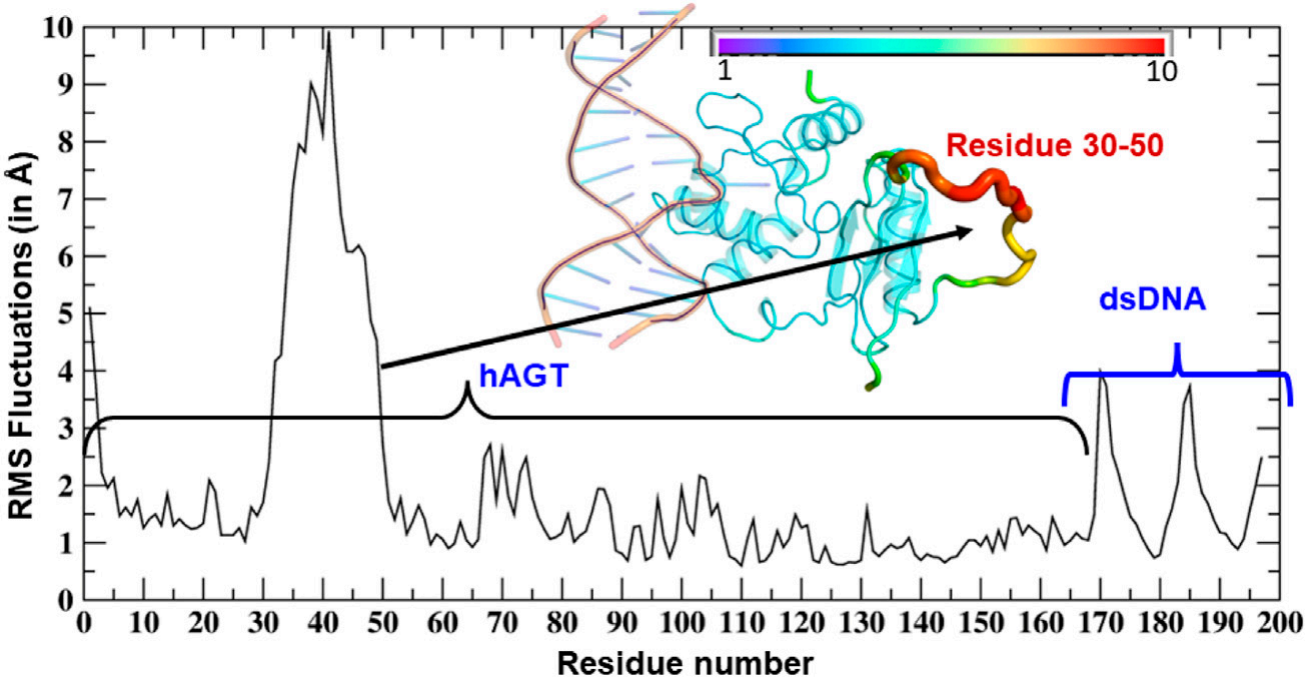
RMSF of dsDNA and the hAGT complex. The thickness of the tube in the inset represents the region of the highest flexibility.

As can be seen (Figure 3), the region of the highest flexibility comes from the residues 30–50 which are from the loop region of hAGT. We note here that this loop region is the zinc-binding region which may have functional significance in the direct repair of the DNA lesion (Mishina et al., 2006). Interestingly, m-GUA (residue 178) shows very small flexibility relative to other DNA regions, which might be due to the strong binding with the catalytic residues of the hAGT enzyme.

**FIGURE 4:**
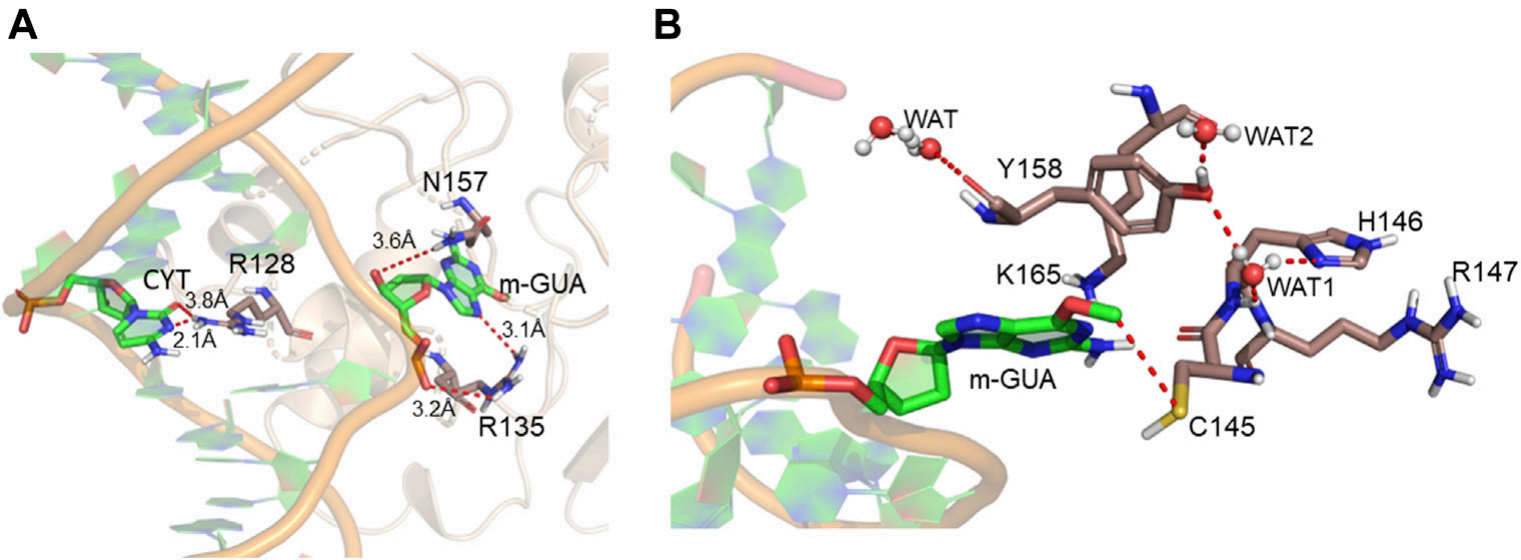
Representative snapshot from the MD simulation. **(A)** Interaction of the DNA bases with the protein. **(B)** Interaction of m-GUA with catalytically important residues.

A representative snapshot from the MD simulation is shown in Figure 4A. As can be seen, R128 (finger residue) occupies the vacant space of guanine and interacts strongly with the orphaned cytosine (Figure 4A). On the other hand, the flipped m-GUA is well installed in the catalytic site and maintains a rigid conformation throughout the entire simulation (Figure 4B). C145 which is supposed to abstract the methyl group of m-GUA resided proximal to m-GUA and maintains proximity with the methylated end. Interestingly, we found a well-organized water chain connecting His146 to Cys145—Tyr158—Lys165 *via* WAT1 and WAT2. The role of His146 has already been proposed during the proton transfer from Cys145 during the de-methylation of m-GUA. To quantify the interaction of m-GUA in the enzymatic site, we calculated the binding free energy of m-GUA into the hAGT enzyme using the molecular mechanics generalized born surface area (MMGBSA) method (Table 1).

**TABLE 1:** Total binding free-energy calculations by the MMGBSA method. All values are in kcal/mol.

Energy component	Average	Std. Dev.
∆E_VDW_	−38.45	3.04
∆E_EEL_	−145.17	7.24
∆E_polar_	154.96	6.16
∆E_nonpolar_	−4.28	0.21
∆G_free_ (TOTAL)	−32.94	4.07

Our calculations show a favorable binding free energy of −32.94 kcal/mol which indicates the twisting of the m-GUA base could be spontaneous and the interactions of the enzymatic site might be the driving force for the twisting.

**FIGURE 5:**
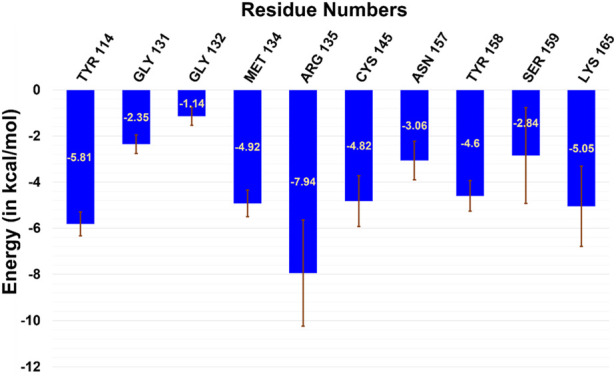
Residue-wise decomposition of the total binding free energy of m-GUA.

Furthermore, to quantify the residue-wise interactions with m-GUA, we calculated the residue-wise decomposition of the total binding free energy using the MMGBSA method (Figure 5). The comprehension of protein–DNA interactions has been greatly aided by the MMGBSA method (Xue et al., 2012; Yang et al., 2018). As can be seen, R135, which is close to the DNA helix, applies the strongest interaction on m-GUA, and therefore, we believe it could be the driving interaction that might lead to the flipping of the methylated DNA base. Furthermore, the catalytic residues, e.g., Tyr114, Cys145, Tyr158, and Lys165 also show significant interactions with m-GUA. Here, it is quite noteworthy that most of the residues which interact with m-GUA are either polar or charged except Met134 which shows that the binding of m-GUA in hAGT is predominantly electrostatically driven.

### Mechanistic elucidation of O6-demethylation *via* QM/MM calculations

**SCHEME 1:**
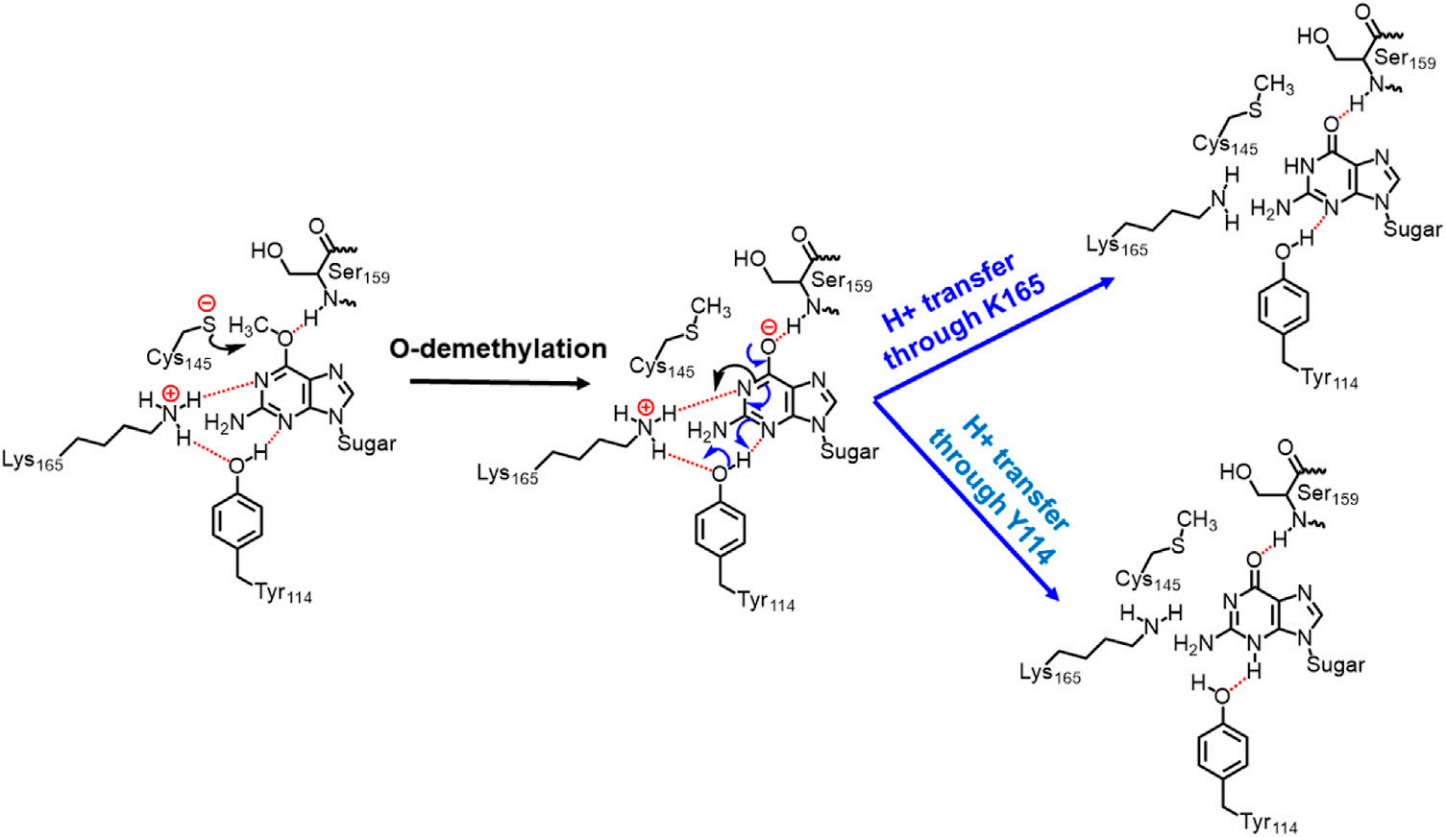
Plausible mechanistic routes for O6-methylguanine repair by AGT. Here, the Cys145 anion acts as a nucleophile and Lys165 and Tyr114 act as H^+^ donors.

In the previous section, we have seen that m-GUA is well installed in the catalytic site of the hAGT enzyme, and it is surrounded by catalytically important residues such as Cy145, Tyr114, Ser159, and Lys165. In addition, we also found a well-organized water channel bridging His146 with Cys145 which can assist the deprotonation of cysteine. The deprotonation mechanism of cysteine *via* histidine is well established, and therefore, we have focused only on the demethylation of m-GUA and formation of guanine since there were discrepancies in the previous investigation of the mechanism (c.f. *Introduction*). For doing so, we employed the QM/MM calculations on a snapshot generated from the 500 ns of MD simulations to study the mechanistic route of DNA repair (O-demethylation of O6-methylguanine) by hAGT. According to prior studies, Cys145 is first deprotonated by His146 through a water molecule, followed by O-demethylation of O6-methylguanine (O6G) by CysSˉ; therefore, we used a deprotonated cysteine during the mechanistic elucidation. A proposed mechanism of the de-methylation of m-GUA is shown in Scheme 1. As can be seen, the anionic charge developed at the “O” atom of guanine following O-demethylation by Cys145 seems to be in resonance with the two “N” atoms, as indicated in the second step of the scheme. Furthermore, Lys165 and Tyr114 are present near the two “N” atoms that can be protonated to regenerate the repaired guanine; we, therefore, investigated the two protonation pathways for demethylated guanine from both Lys165 and Tyr114.

**SCHEME 2:**
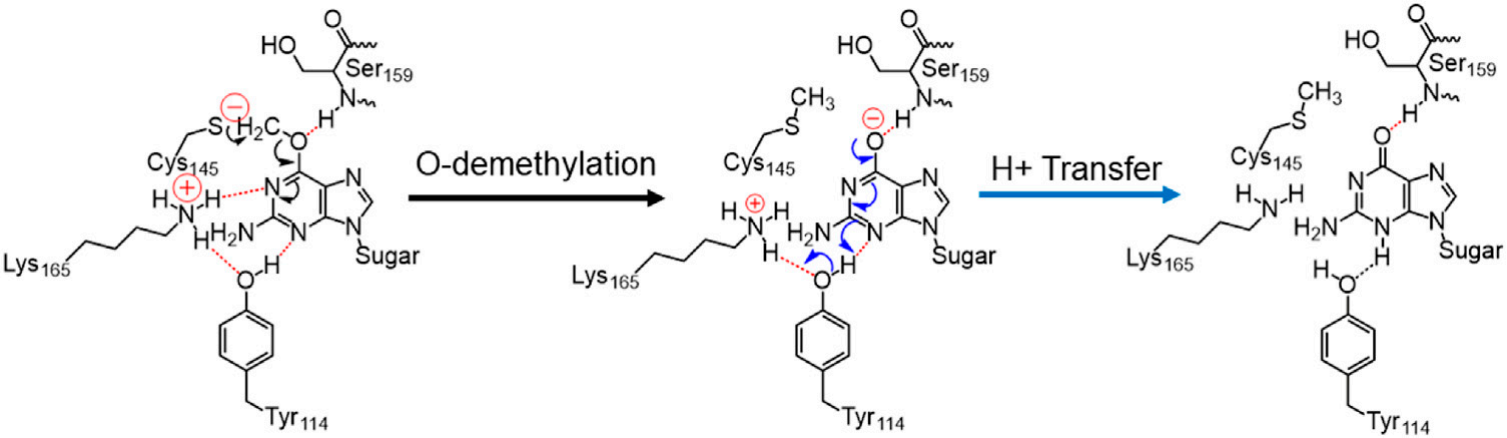
Final mechanism of direct repair of m-GUA by hAGT.

**FIGURE 6:**
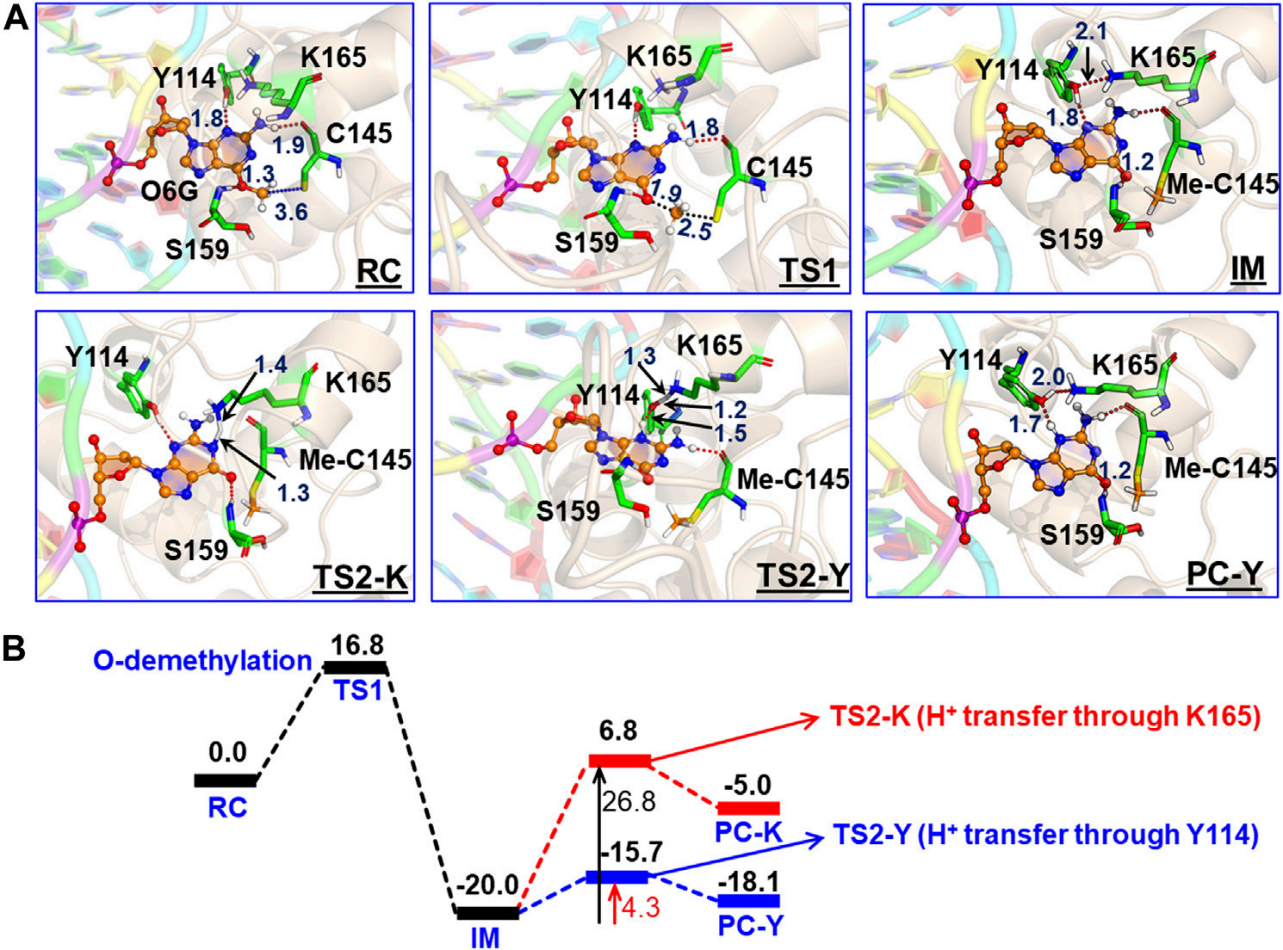
**(A)** QM/MM-optimized geometries along with the key geometric data for RC, TS1, TS2-Y, TS2-K, IM, and PC-Y. The geometry for PC-K can be found in SI. “Me-C145” represents the methylated Cys145. **(B)** Complete reaction profile diagram. The energy values (reported in kcal/mol) are noted for the optimized structures (B3LYP/def2-SVP) of all the RC, TS, IM, and PC states. All energies are corrected by zero-point energy (ZPE) and Grimme’s dispersion (G-D3).

To get a reactant cluster (RC), we picked a representative snapshot from the MD trajectories based on the most populated structure and performed QM/MM geometry optimization. In the optimized RC, the methyl carbon of m-GUA was seen to be 3.6 Å distant from the CysSˉ nucleophile (Figure 6A). To acquire the whole reaction route, we performed relaxed potential energy surface (PES) scanning, and the reaction profile is shown in Figure 6B. In the first step of the reaction, CysSˉ attacks the methyl carbon to perform the O-demethylation of O6-methylguanine through a transition state (TS) barrier of 16.8 kcal/mol (see TS1, Figure 6A), followed by the formation of anionic guanine as intermediate IM.

After the formation of anionic guanine, it needs to be protonated to generate the repaired guanine. As discussed earlier, it could be *via* two pathways: either *via* the Lys165 or Tyr114 routes. Therefore, we explored both routes through PES scanning, and the reaction profile for the same is shown in blue and red in Figure 6B. The reaction profile in red depicts the relative transition state barrier for H^+^ transfer to guanine *via* Lys165 while the profile in blue shows the H^+^ transfer to guanine *via* Tyr114. As can be seen, the TS barrier for H^+^ transfer from Lys165 is observed to be 26.8 kcal/mol which is quite a high barrier for H^+^ transfer reactions in enzymatic reactions. On the other hand, the production of the repaired guanine *via* H^+^ transfer by Tyr114 is very facile and occurs through a low barrier of 4.3 kcal/mol. Therefore, the second route is relatively preferable over Lys165 for the protonation of demethylated guanine. Interestingly, we found that as soon as the H^+^ is transferred from Tyr114 onto guanine, one H^+^ is retrieved from Lys165 to rejuvenate itself. Furthermore, we found that Ser159 which is close to m-GUA stabilizes the TS and plays a crucial role in the catalysis.

In a nutshell, we can state that this entire DNA repair mechanism (demethylation of O6-methylguanine) consists of three steps (Scheme 2): first, Cys145 is deprotonated to function as a nucleophile, then it performs the O-demethylation, and finally, H^+^ transfer occurs from Tyr114 to form the repaired guanine.

## Conclusion

In the present study, using comprehensive MD simulation of the double-stranded DNA in complex with the hAGT enzyme and hybrid QM/MM calculations, we have studied the mechanism of direct DNA repair by the hAGT enzyme. Our MD simulations show that methylated guanine has several favorable interactions with the protein residues, particularly, Arg135 that provides the driving force for base flipping. Furthermore, the flipped base is thermodynamically stabilized by several polar and charged residues in the active site. The QM/MM study reveals the mechanism of the demethylation by the Cys145 residue, and we show that a complete repair of the guanine can be formed *via* Tyr114 rather than Lys165. In addition, our reaction profile shows irreversible repairing which is in good agreement with the proposed suicidal and irreversible repairing by hAGT enzymes.

## Data Availability

The original contributions presented in the study are included in the article/Supplementary Material; further inquiries can be directed to the corresponding author.
