# Supplementary material for: Unraveling key interactions and the mechanism of demethylation during hAGT-mediated DNA repair via simulations
Source: Front Mol Biosci. 2022 Sep 14;9:975046. doi: 10.3389/fmolb.2022.975046 (PMC9515978; doi:10.3389/fmolb.2022.975046)
Supplement: Supplementary file 1 [file DataSheet1.PDF]

Supporting Information

Unraveling Key Interactions and the Mechanism of Demethylation during hAGT mediated DNA Repair via Simulations

Shruti T G†[1], Shakir Ali Siddiqui, †[2] and Kshatresh Dutta Dubey*[2]

1. Department of Life Sciences, School of Natural Sciences, Shiv Nadar University Delhi-NCR, NH-91, Gautam Buddha Nagar, Uttar Pradesh-201314, India
2. Department of Chemistry, School of Natural Sciences, Shiv Nadar University Delhi-NCR, NH-91, Gautam Buddha Nagar, Uttar Pradesh-201314, India

†Authors have Equal Contributions.

* Corresponding author, email: kshatresh.dubey@snu.edu.in

Table of Contents:

1. H-Bonds Analysis between DNA and protein ……………………………S2
2. H Bond between Cys145 and damaged Guanine …………………………S3
3. Figure S1. The QM/MM optimized geometry of PC-K ……………..…..S3
4. Table S1. pKa of residues at the protein-ligand interface…………………S4
5. QM Geometry for QM/MM Optimized Structures ………………….…S4-S10

H-Bonds Analysis between DNA and protein: H bonds were calculated by uploading 200ns trajectory in VMD, here 10 ps is one frame.

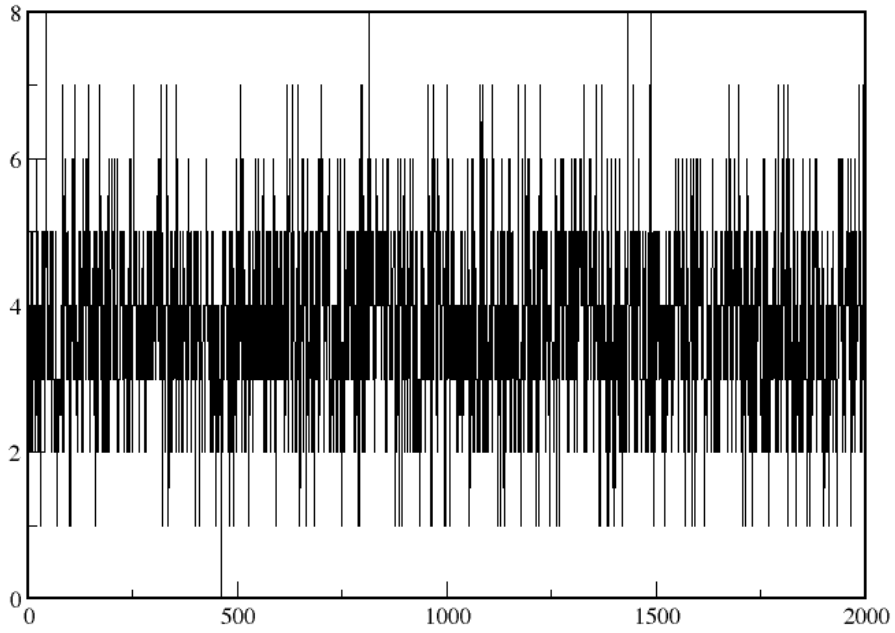

Figure depicts the H-bond analysis for DNA-Protein interaction, with no solvent Consideration. This was performed over 200 ns of simulation time. There is high density of ¾/5 (maximum 4, thickest band on graph) Hydrogen bonds between DNA and the protein regions of the complex. Some of the major interactions are –

O6G178side	CYS141Main	47.20%
THR91side	DT194side	68.35%
SER147side	DT180side	87.05%
TYR110side	O6G178side	60.60

These interactions were also proposed to play significant role in the stability of the extra helical damaged base and prevent its untwisting.

H Bond between Cys145 and damaged Guanine: H bonds were calculated by uploading 200ns trajectory in VMD, here 10 ps is one frame.

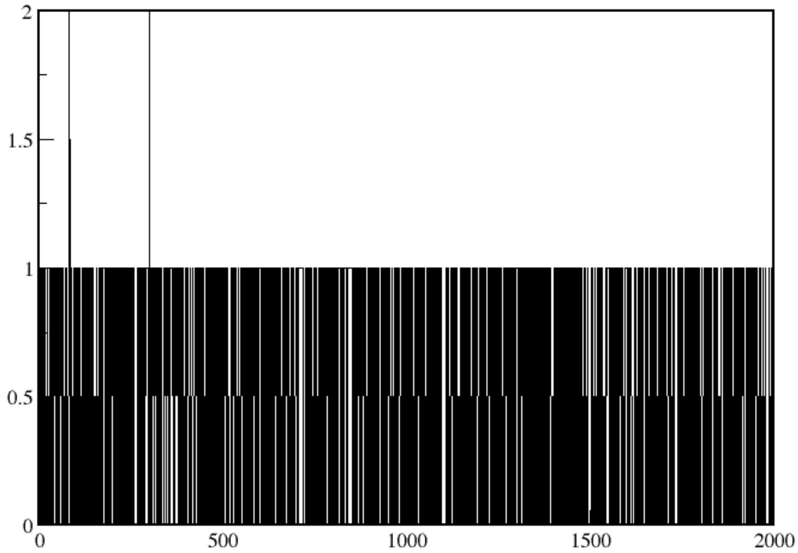

Between the active site residue – Cys145 and the dna, there is almost no instance where HB formations don't take place. There are HB bonds formed over the entire 200ns, indicating a strong affinity between the O6G substrate and Cys residue- both for ensuring the modified base stays in vicinity of the active site, as well as to mediate the alkyl removal by nucleophilic attack.

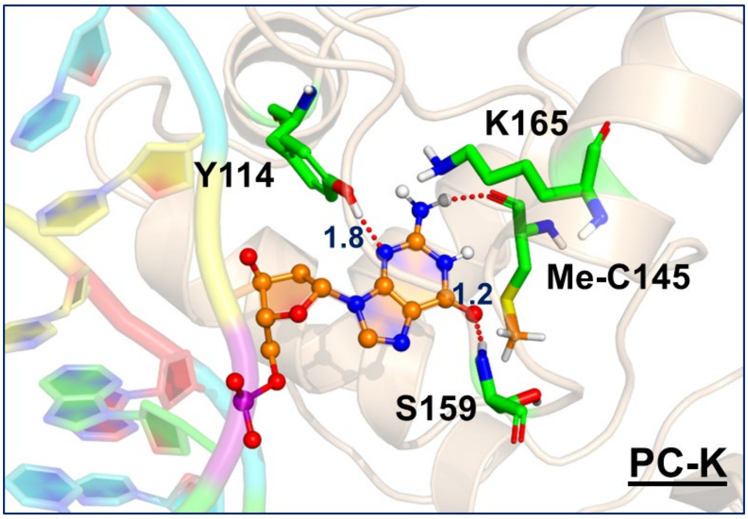

Figure S1. The QM/MM optimized geometry of PC-K (product formed through H^+ transfer from Lys165). Distances are in Å.

Table S1. pKa of residues at the protein-ligand interface

Residues	pKa
Y114	9.59
R135	12.50
Y158	10.00
K165	10.50

QM Geometry for QM/MM Optimized Structures:

RC

C -11.6745291 0.0857268 11.8147208

H -12.0679311 -0.0963382 12.8259640

H -12.5189263 -0.0220033 11.1110911

C -10.5880143 -0.9235625 11.4889035

C -9.9845242 -1.6938986 12.4965413

H -10.3439347 -1.5962578 13.5238668

C -8.9354111 -2.5775375 12.2231387

H -8.4796193 -3.1630228 13.0248418

C -8.4626044 -2.7206328 10.9144737

O -7.3930895 -3.5434831 10.6617496

H -7.4011547 -3.8190238 9.7112953

C -9.1043535 -2.0161359 9.8834431

H -8.7863385 -2.1764468 8.8517917

C -10.1494682 -1.1370587 10.1719416

H -10.6229897 -0.5982142 9.3462273

C -4.0288622 -0.7771437 4.4414573

H -3.0519971 -1.0623361 3.9936804

H -4.3580906 -1.6308385 5.0607686

S -5.3231113 -0.3379424 3.2286377

C -4.3064880 -7.5476450 2.0184130

H -4.5241608 -8.1991813 1.1502046

H -5.2259598 -6.9909574 2.2727992

O -3.2084855 -6.6830079 1.7582894

H -3.5121005 -6.1155568 1.0031016

C -0.5843298 -4.1499681 9.9815016

H -0.1852582 -3.8617743 10.9674760

H -0.3389727 -3.3309095 9.2890948

C -2.1019068 -4.3770630 10.0570458

H -2.4968286 -4.6410050 9.0632345

H -2.3212013 -5.2316497 10.7180726

C -2.9206585 -3.1785420 10.5307630

H -2.5381037 -2.7410739 11.4633731

H -2.9591831 -2.3750113 9.7819662

N -4.3365786 -3.6038641 10.7955134

H -4.9898052 -2.7972865 10.7588930

H -4.6705249 -4.3267210 10.1295153

H -4.4076532 -4.0010363 11.7517938

N -8.2721119 -6.3282526 7.5038966

C -7.3985170 -5.3035209 7.2138047

N -7.0896463 -4.2188151 7.9315008

C -6.1318138 -3.4574102 7.3616528

N -5.6678744 -2.4019678 8.0822049

N -5.5640019 -3.6746995 6.1522760

C -5.9276307 -4.7268920 5.4337406

O -5.4057929 -4.9692818 4.2377200

C -6.8573903 -5.6518136 5.9656051

N -7.3616226 -6.8570387 5.5196479

C -8.1953061 -7.2369725 6.4446178

C -4.6905884 -3.9255069 3.5401648

H -6.1871706 -2.0904972 8.8978506

H -5.0690139 -1.7039815 7.6405460

H	-8.8000504	-8.1454823	6.4290783
H	-4.3909303	-4.3838018	2.5910402
H	-5.3205745	-3.0316029	3.3684557
H	-3.7996618	-3.6343780	4.1101216
H	-11.3305884	1.1190442	11.7696992
H	-3.8232139	0.0589131	5.1098771
H	-4.1218493	-8.1945041	2.8760545
H	-0.0606374	-5.0414603	9.6364580
H	-8.8085016	-6.4414446	8.3402255

TS1

C	-11.6740957	0.0863987	11.8069719
H	-12.0684263	-0.0977585	12.8176234
H	-12.5188517	-0.0186267	11.1031566
C	-10.5898190	-0.9250489	11.4792514
C	-9.9818594	-1.6890818	12.4891452
H	-10.3349193	-1.5829549	13.5178760
C	-8.9380526	-2.5778761	12.2161293
H	-8.4799937	-3.1598537	13.0191202
C	-8.4713911	-2.7356033	10.9055981
O	-7.4100007	-3.5639609	10.6600961
H	-7.4070390	-3.8511529	9.7070092
C	-9.1181648	-2.0359304	9.8725922
H	-8.8078050	-2.2070596	8.8397224
C	-10.1590049	-1.1510199	10.1616563
H	-10.6378705	-0.6192411	9.3341889
C	-3.5290010	-1.0160003	4.3400012
H	-2.4799059	-1.1411771	4.0188371
H	-3.7976115	-1.8824931	4.9706334
S	-4.6930013	-0.9520003	2.9470008
C	-4.2930107	-7.4838219	2.0558821
H	-4.5589253	-8.1141643	1.1840559
H	-5.1796117	-6.8960423	2.3528485
O	-3.1730265	-6.6559353	1.7729713
H	-3.4879342	-6.0610212	1.0489830
C	-0.5918405	-4.1720212	10.0050960
H	-0.2021645	-3.9050344	11.0009403
H	-0.3397347	-3.3387677	9.3327203
C	-2.1101598	-4.4009320	10.0581687
H	-2.4942141	-4.6481757	9.0555544
H	-2.3380411	-5.2657729	10.7029838
C	-2.9310828	-3.2080469	10.5429773
H	-2.5529553	-2.7830135	11.4832121
H	-2.9630775	-2.3958194	9.8036861
N	-4.3493210	-3.6299928	10.7918960
H	-4.9989619	-2.8222198	10.7398826
H	-4.6761787	-4.3516047	10.1203039
H	-4.4329239	-4.0222616	11.7484687
N	-8.2921399	-6.3681734	7.5394404
C	-7.3918296	-5.3623867	7.2577328
N	-7.0811730	-4.2764329	7.9829808
C	-6.1008789	-3.5410677	7.4147129
N	-5.6237315	-2.4822908	8.1482096
N	-5.5166851	-3.7686409	6.2290048
C	-5.8670016	-4.8350014	5.4660015
O	-5.3420015	-5.0280014	4.3090012
C	-6.8400677	-5.7236720	6.0211169
N	-7.3667638	-6.9192685	5.5711994
C	-8.2201364	-7.2824517	6.4849046
C	-5.0040014	-3.3890010	3.4260010
H	-6.1911121	-2.1278903	8.9129131
H	-5.0398395	-1.7881254	7.6843063
H	-8.8433513	-8.1778511	6.4646073
H	-5.4900015	-3.6780010	2.4990007

H	-5.5530016	-2.8470008	4.1740012
H	-3.9250011	-3.4710010	3.4920010
H	-11.3306921	1.1200437	11.7655078
H	-3.5671055	-0.1338538	4.9790887
H	-4.1113962	-8.1522699	2.8974643
H	-0.0627922	-5.0546553	9.6457466
H	-8.8307144	-6.4732834	8.3754199

<u>IM</u>

C	-11.6766312	0.0970488	11.8165739
H	-12.0756132	-0.0820295	12.8263494
H	-12.5186894	-0.0090981	11.1096451
C	-10.5923438	-0.9161350	11.4995671
C	-9.9824069	-1.6678473	12.5173810
H	-10.3353149	-1.5498424	13.5448433
C	-8.9398001	-2.5595570	12.2529402
H	-8.4806476	-3.1332429	13.0612539
C	-8.4741257	-2.7343254	10.9429679
O	-7.4229382	-3.5694936	10.7064620
H	-7.3857814	-3.8216793	9.7369181
C	-9.1219930	-2.0420480	9.9032211
H	-8.8172221	-2.2311180	8.8721318
C	-10.1625826	-1.1546730	10.1844491
H	-10.6447664	-0.6322065	9.3523107
C	-3.7595443	-1.2401504	4.4697248
H	-2.7416126	-1.4067353	4.0801501
H	-4.0610636	-2.0766446	5.1298065
S	-4.9510711	-1.2152150	3.0933754
C	-4.2946090	-7.4178051	2.1021909
H	-4.6394075	-8.0237674	1.2406214
H	-5.1289310	-6.7881028	2.4582892
O	-3.1541197	-6.6428004	1.7546992
H	-3.4851145	-6.0279208	1.0581305
C	-0.5938676	-4.1697154	9.9945715
H	-0.1972050	-3.8707857	10.9784797
H	-0.3563893	-3.3535425	9.2956437
C	-2.1091491	-4.4158889	10.0688150
H	-2.4984340	-4.7103723	9.0813666
H	-2.3196740	-5.2574859	10.7489873
C	-2.9415702	-3.2148409	10.5125568
H	-2.5633730	-2.7481327	11.4329889
H	-2.9884236	-2.4350816	9.7397695
N	-4.3544486	-3.6368745	10.7812856
H	-5.0067953	-2.8351644	10.6891078
H	-4.6767921	-4.3828465	10.1335820
H	-4.4363856	-3.9926906	11.7517259
N	-8.2196143	-6.2459535	7.5011199
C	-7.3160415	-5.2240502	7.2768335
N	-7.0403578	-4.1556447	8.0484571
C	-6.0371459	-3.4038436	7.5356437
N	-5.6370573	-2.3422476	8.3202933
N	-5.3604399	-3.6052233	6.4045351
C	-5.6192811	-4.7014731	5.6050270
O	-4.9263687	-4.9458544	4.5948078
C	-6.6961152	-5.5515241	6.0703917
N	-7.1858734	-6.7476223	5.5754017
C	-8.0832280	-7.1392680	6.4356420
C	-4.4703717	-2.8542445	2.4404953
H	-6.2835850	-1.9895468	9.0190677
H	-5.0374312	-1.6351358	7.8995730
H	-8.6972228	-8.0387808	6.3661723
H	-5.1659629	-3.1436112	1.6435052
H	-4.5358610	-3.6090457	3.2437878
H	-3.4449174	-2.7972696	2.0462882

H	-11.3290814	1.1291474	11.7713100
H	-3.7474123	-0.3112726	5.0399055
H	-4.1026757	-8.1071518	2.9244012
H	-0.0587554	-5.0552413	9.6516925
H	-8.7705328	-6.3886102	8.3233791

TS2-K

C	-11.8172852	0.1505567	11.9335395
H	-12.2033768	0.0445050	12.9567960
H	-12.6796703	0.0634185	11.2486849
C	-10.8224170	-0.9419287	11.6520507
C	-10.2328090	-1.6749236	12.6936307
H	-10.5255169	-1.4566702	13.7211336
C	-9.2887411	-2.6689298	12.4480613
H	-8.8363127	-3.2326147	13.2657829
C	-8.9137892	-2.9640063	11.1315530
O	-7.9674483	-3.9097506	10.9158849
H	-7.9002961	-4.0681373	9.9478709
C	-9.5359201	-2.2696283	10.0737926
H	-9.3191952	-2.5481171	9.0426085
C	-10.4755771	-1.2773693	10.3405896
H	-10.9553287	-0.7638477	9.5018047
C	-3.6171482	-1.4228008	4.4720316
H	-2.6008074	-1.5066895	4.0526394
H	-3.8062081	-2.2840484	5.1361355
S	-4.8697615	-1.5048340	3.1524741
C	-4.3102294	-7.2730972	2.1840544
H	-4.6579491	-7.8711904	1.3197485
H	-5.1562509	-6.6568844	2.5394997
O	-3.1730841	-6.4923813	1.8416811
H	-3.4752486	-5.9545942	1.0693044
C	-0.5511894	-4.1881875	10.0485538
H	-0.2508995	-3.9910876	11.0902476
H	-0.2257825	-3.3186904	9.4550991
C	-2.0652219	-4.3957424	9.9217520
H	-2.3106593	-4.6715383	8.8862326
H	-2.3963725	-5.2430992	10.5453527
C	-2.9375683	-3.1872946	10.2392581
H	-3.0921828	-3.0856679	11.3267340
H	-2.4830993	-2.2428042	9.9009544
N	-4.2544968	-3.3317765	9.5729060
H	-4.8790393	-2.6350112	10.0001341
H	-4.5944905	-3.2912034	8.2038187
H	-4.6486720	-4.2589696	9.7986218
N	-8.2943727	-6.2305178	7.4115544
C	-7.5164821	-5.0973031	7.3709805
N	-7.6236940	-3.9993569	8.1401688
C	-6.6139909	-3.1477863	7.9746543
N	-6.6707068	-1.9483261	8.5846948
N	-5.4703715	-3.3947012	7.2641990
C	-5.4682560	-4.4003723	6.2791707
O	-4.5356938	-4.4948961	5.4679190
C	-6.5936063	-5.3076963	6.3542902
N	-6.8034675	-6.5453873	5.7649498
C	-7.8022264	-7.0782291	6.4174290
C	-4.5187884	-3.2244446	2.6392678
H	-7.3475144	-1.7925982	9.3263532
H	-5.8594434	-1.3370149	8.5520547
H	-8.2508200	-8.0550889	6.2308941
H	-5.2347395	-3.5016647	1.8556599
H	-4.6455637	-3.8899903	3.5044294
H	-3.4888810	-3.3121363	2.2632565
H	-11.3943566	1.1482844	11.8163304
H	-3.6794541	-0.4897529	5.0320376

H	-4.1090792	-7.9709371	2.9968430
H	-0.0168672	-5.0617177	9.6750411
H	-8.8976077	-6.4512616	8.1779433

PC-K

C	-11.7483551	0.1346660	11.8681535
H	-12.1481691	-0.0045432	12.8832378
H	-12.5978022	0.0386891	11.1679015
C	-10.7098596	-0.9235049	11.5802901
C	-10.1508020	-1.6852247	12.6185906
H	-10.5003026	-1.5231961	13.6400625
C	-9.1640056	-2.6397745	12.3783329
H	-8.7346807	-3.2236164	13.1944951
C	-8.7075295	-2.8642578	11.0737881
O	-7.7200075	-3.7746690	10.8755410
H	-7.6412257	-3.9829901	9.9199206
C	-9.2927491	-2.1438539	10.0161173
H	-8.9850698	-2.3481071	8.9894881
C	-10.2805238	-1.1947826	10.2743601
H	-10.7224400	-0.6517079	9.4327708
C	-3.5846507	-1.3502365	4.2623482
H	-2.5940862	-1.3798812	3.7801950
H	-3.6932878	-2.2647496	4.8655827
S	-4.9306849	-1.3864993	3.0374804
C	-4.3053356	-7.3796147	2.1739448
H	-4.6357440	-7.9900308	1.3111006
H	-5.1650549	-6.7826467	2.5273405
O	-3.1884957	-6.5697944	1.8306796
H	-3.5167512	-6.0022853	1.0923326
C	-0.4645633	-4.1892260	10.1924389
H	-0.1464138	-4.1966877	11.2487802
H	-0.0724863	-3.2543127	9.7571613
C	-1.9905897	-4.2294433	10.0845841
H	-2.3032664	-4.1561053	9.0261202
H	-2.3868258	-5.1931527	10.4508353
C	-2.7094770	-3.1068687	10.8277672
H	-2.5721563	-3.2441894	11.9196925
H	-2.2445465	-2.1357553	10.5763018
N	-4.1062563	-3.0778892	10.4121266
H	-4.6091876	-2.3263170	10.8946738
H	-4.7723649	-2.9487973	6.5037753
H	-4.5663625	-3.9388394	10.7184340
N	-8.3050634	-6.2898126	7.4650913
C	-7.4622074	-5.2198526	7.2921694
N	-7.3338846	-4.1452645	8.0910310
C	-6.3527938	-3.3233605	7.7533160
N	-6.1371330	-2.1703141	8.3991945
N	-5.5502971	-3.5802881	6.6627960
C	-5.6436357	-4.6743372	5.7823326
O	-4.7952987	-4.8119318	4.8942809
C	-6.7402691	-5.5234250	6.1305233
N	-7.1249215	-6.7454623	5.6142349
C	-8.0496879	-7.1814889	6.4228527
C	-4.5323502	-3.0369583	2.3584957
H	-6.5048646	-2.0750734	9.3483168
H	-5.2076366	-1.7501246	8.3285911
H	-8.6032448	-8.1170798	6.3283303
H	-5.2357103	-3.2588606	1.5470762
H	-4.6427535	-3.7970754	3.1437868
H	-3.5022329	-3.0359491	1.9777979
H	-11.3610667	1.1503122	11.7872987
H	-3.6328047	-0.4620493	4.8923257
H	-4.0903715	-8.0688707	2.9905131
H	0.0020915	-5.0334351	9.6848844

H -8.8579320 -6.4577609 8.2812421

TS2-Y

C -11.5048719 0.0462201 11.8109031
H -11.8800567 -0.1783765 12.8210958
H -12.3454396 -0.1098285 11.1109014
C -10.3468048 -0.8889280 11.4560862
C -9.7395851 -1.7277379 12.4079848
H -10.1398496 -1.7443980 13.4253338
C -8.6346159 -2.5404874 12.1042044
H -8.2015252 -3.1794144 12.8784430
C -8.0833353 -2.5542786 10.8108724
O -7.0376495 -3.3486070 10.4371680
H -7.1617538 -3.9267285 8.9674402
C -8.7380132 -1.7722255 9.8351337
H -8.3650721 -1.8046503 8.8122608
C -9.8383257 -0.9726192 10.1477915
H -10.2992907 -0.3837002 9.3472693
C -3.6731802 -1.1792897 4.4265788
H -2.6548794 -1.3350807 4.0350875
H -3.9732320 -2.0224057 5.0757564
S -4.8717479 -1.1577609 3.0568755
C -4.2430276 -7.4377012 2.1085550
H -4.5683035 -8.0520882 1.2454278
H -5.0940461 -6.8249864 2.4535923
O -3.1161620 -6.6399685 1.7706557
H -3.4546676 -6.0232938 1.0801181
C -0.8190434 -4.2354654 9.6714116
H -0.2606876 -3.4038578 10.1345725
H -0.9883701 -3.9661635 8.6124720
C -2.1687734 -4.4475815 10.4189682
H -2.5559031 -5.4653593 10.2522447
H -1.9804187 -4.3917342 11.5002119
C -3.3303652 -3.4677465 10.1104014
H -3.0982302 -2.4415413 10.4374843
H -3.5235449 -3.4292466 9.0263897
N -4.5967912 -3.8922511 10.7802989
H -5.8341229 -3.3794266 10.6703707
H -4.7844536 -4.8653503 10.4911057
H -4.4336904 -3.9277471 11.8003767
N -8.1282185 -6.2606085 7.5335462
C -7.2771488 -5.2284800 7.2329085
N -6.9719447 -4.1018482 7.9276486
C -6.0285594 -3.2778357 7.3503142
N -5.7672025 -2.1428638 8.0308826
N -5.4174399 -3.5291344 6.2049607
C -5.6562972 -4.6860875 5.4833813
O -4.9761469 -4.9767686 4.4885793
C -6.7053431 -5.5464638 6.0118275
N -7.1720242 -6.7699687 5.5707426
C -8.0140749 -7.1741346 6.4772121
C -4.3880608 -2.7964041 2.4071189
H -6.0532007 -2.0432197 9.0055961
H -5.0098867 -1.5351509 7.7272388
H -8.6057226 -8.0903895 6.4536676
H -5.0607637 -3.0734564 1.5865949
H -4.4804082 -3.5602392 3.1965279
H -3.3513014 -2.7451018 2.0432626
H -11.2421619 1.1037571 11.7850924
H -3.6674185 -0.2562936 5.0063307
H -4.0456102 -8.1228274 2.9329915
H -0.1708499 -5.1113891 9.6455284
H -8.6694448 -6.3900159 8.3643893

PC-Y

C	-11.6510545	0.0867775	11.8349306
H	-12.0431343	-0.0950714	12.8462784
H	-12.4924501	-0.0382191	11.1300677
C	-10.5440364	-0.9022793	11.5131909
C	-10.0106780	-1.7519697	12.4958741
H	-10.4357382	-1.7338659	13.5025599
C	-8.9414277	-2.6153646	12.2213410
H	-8.5457870	-3.2718617	12.9994991
C	-8.3774817	-2.6368447	10.9441605
O	-7.3475707	-3.5063217	10.6117885
H	-7.2471440	-3.9903454	8.9682193
C	-8.9400486	-1.8451487	9.9357371
H	-8.5186768	-1.8938737	8.9322098
C	-10.0125543	-1.0012658	10.2163439
H	-10.4268448	-0.3890546	9.4094735
C	-3.7007529	-1.1947160	4.4295759
H	-2.6812710	-1.3561117	4.0436902
H	-4.0069188	-2.0351112	5.0789913
S	-4.8902144	-1.1639676	3.0523208
C	-4.2457973	-7.4198416	2.1204228
H	-4.5750559	-8.0354807	1.2597357
H	-5.0954482	-6.8061523	2.4672655
O	-3.1193256	-6.6242838	1.7765048
H	-3.4575695	-6.0139548	1.0802131
C	-0.7275060	-4.2247493	9.6530526
H	-0.1486620	-3.4040469	10.1111515
H	-0.9005917	-3.9536716	8.5945269
C	-2.0622805	-4.4201949	10.4077133
H	-2.4642731	-5.4370341	10.2655029
H	-1.8735838	-4.3406112	11.4877384
C	-3.2057445	-3.4392999	10.0759032
H	-2.9308306	-2.4111880	10.3719855
H	-3.3667140	-3.4191247	8.9814825
N	-4.4738323	-3.8002902	10.7238365
H	-6.4249595	-3.2278569	10.8606250
H	-4.7084795	-4.7590412	10.4356565
H	-4.3228496	-3.8420286	11.7419414
N	-8.1592293	-6.2828727	7.5398173
C	-7.3115721	-5.2458461	7.2517732
N	-7.0135421	-4.1209349	7.9581379
C	-6.0578452	-3.2910861	7.4039464
N	-5.8048368	-2.1536936	8.0812340
N	-5.4460231	-3.5348009	6.2586937
C	-5.6805913	-4.6819268	5.5202577
O	-4.9984697	-4.9591138	4.5240612
C	-6.7304334	-5.5506958	6.0337308
N	-7.1906518	-6.7712208	5.5788739
C	-8.0367831	-7.1867631	6.4762184
C	-4.4186024	-2.8083924	2.4076517
H	-6.0149053	-2.0547697	9.0763975
H	-5.0297070	-1.5715724	7.7688413
H	-8.6249437	-8.1049230	6.4422457
H	-5.0946659	-3.0844410	1.5895525
H	-4.5156341	-3.5643636	3.2034269
H	-3.3815446	-2.7680791	2.0440181
H	-11.3181053	1.1232970	11.7816445
H	-3.6930308	-0.2714552	5.0088832
H	-4.0457124	-8.1042253	2.9448330
H	-0.1081744	-5.1213098	9.6270762
H	-8.7112094	-6.4145201	8.3632001
